# Supplementary material for: Bilateral ankle dorsiflexion force control impairments in older adults
Source: PLoS One. 2025 Mar 20;20(3):e0319578. doi: 10.1371/journal.pone.0319578 (PMC11925285; doi:10.1371/journal.pone.0319578)
Supplement: S1 Table — MVC = maximum voluntary contraction, rRMSE = relative root mean square error, rBE- relative bias error, %CV = coefficient of variation, rcMSE = refined composite multiscale sample entropy. Data are mean ± SD. Number sign (#) means a significant difference between force levels. Ampersand sign (&) denotes a significant difference between vision conditions. (P < 0.05). (DOCX) [file pone.0319578.s001.docx]

**S1 Table. Bilateral force control capabilities for the younger and older groups.**

| **Variable** | **Significance** | **Vision condition** | **Force level** | |
| --- | --- | --- | --- | --- |
|  |  |  | **10% MVC** | **40% MVC** |
| rRMSE (%Target) | Group × Force Level × Vision Condition interaction | Vision | Young: 1.6 ± 0.6^#&^  Old: 2.9 ± 1.2^&^ | Young: 2.1 ± 0.8^#&^  Old: 2.6 ± 1.1^&^ |
|  |  | No-vision | Young: 6.1 ± 2.9^&^  Old: 11.5 ± 5.3^#&^ | Young: 8.0 ± 3.0^&^  Old: 8.1 ± 4.1^#&^ |
| rBE (%Target) | Group × Force Level × Vision Condition interaction | Vision | Young: 0.3 ± 0.7^#^  Old: 1.3 ± 1.6^#&^ | Young: −1.5 ± 0.8^#&^  Old: −1.3 ± 1.7^#&^ |
|  |  | No-vision | Young: 1.8 ± 3.9^#^  Old: 8.5 ± 7.0^#&^ | Young: −6.7 ± 3.2^#&^  Old: −5.4 ± 6.0^#&^ |
| %CV | Group × Force Level × Vision Condition interaction | Vision | Young: 1.3 ± 0.4^&^  Old: 1.9 ± 1.0^#&^ | Young: 1.3 ± 0.5^&^  Old: 1.6 ± 0.6^#&^ |
|  |  | No-vision | Young: 3.0 ± 1.6^#&^  Old: 4.2 ± 1.5^&^ | Young: 3.8 ± 1.5^#&^  Old: 3.5 ± 1.6^&^ |
| rcMSE  (force complexity) | Group × Force Level × Vision Condition interaction | Vision | Young: 7.7 ± 1.4^&^  Old: 6.8 ± 1.5^&^ | Young: 7.6 ± 1.4^&^  Old: 6.6 ± 1.5^&^ |
|  |  | No-vision | Young: 4.4 ± 1.2^#&^  Old: 3.4 ± 1.3^&^ | Young: 3.2 ± 1.3^#&^  Old: 3.3 ± 1.2^&^ |

*Abbreviations*. MVC = maximum voluntary contraction, rRMSE = relative root mean square error, rBE- relative bias error, %CV = coefficient of variation, rcMSE = refined composite multiscale sample entropy. Data are mean ± SD. Number sign (#) means a significant difference between force levels. Ampersand sign (&) denotes a significant difference between vision conditions. (*P* < 0.05).
