# Supplementary material for: Bilateral ankle dorsiflexion force control impairments in older adults
Source: PLoS One. 2025 Mar 20;20(3):e0319578. doi: 10.1371/journal.pone.0319578 (PMC11925285; doi:10.1371/journal.pone.0319578)
Supplement: S2 Table — Data are mean ± SD. Number sign (#) means a significant difference between force levels. Ampersand sign (&) denotes a significant difference between vision conditions. (P < 0.05). (DOCX) [file pone.0319578.s002.docx]

**S2 Table. Bilateral force coordination for the younger and older groups.**

| **Variable** | **Significance** | **Vision Condition** | **Force Level** | |
| --- | --- | --- | --- | --- |
|  |  |  | **10% MVC** | **40% MVC** |
| In-phase frequency | Group × Force Level interaction | - | Young: 239.4 ± 75.1^#^  Old: 222.6 ± 68.8^#^ | Young: 383.3 ± 51.9^#^  Old: 403.3 ± 66.8^#^ |
|  | Force Level × Vision Condition interaction | Vision | 232.5 ± 75.6^#^ | 412.4 ± 61.6^#&^ |
|  |  | No-vision | 229.6 ± 69.2^#^ | 374.2 ± 53.1^#&^ |
| Anti-phase frequency | Group × Force Level interaction | - | Young: 151.1 ± 26.0^#^  Old: 142.7 ± 28.5^#^ | Young: 224.8 ± 32.1^#^  Old: 187.5 ± 43.2^#^ |
|  | Force Level × Vision Condition interaction | Vision | 140.2 ± 22.7^#&^ | 195.3 ± 41.1^#&^ |
|  |  | No-vision | 153.7 ± 30.3^#&^ | 217.0 ± 41.0^#&^ |
| V_Index_ (Z-transform) | Group× Vision Condition interaction | Vision | Young: 2.2 ± 0.7^&^  Old: 2.1 ± 0.7^&^ | |
|  |  | No-vision | Young: 0.3 ± 0.6^&^  Old: 0.2 ± 0.5^&^ | |
| V_UCM_ | Force Level main effect | - | 72.1 ± 107.9 | 15.2 ±17.2 |
| V_ORT_ | Group main effect | - | Young: 5.7 ± 11.2  Old: 8.9 ± 13.4 | |
|  | Force Level × Vision Condition interaction | Vision | 0.5 ± 0.6^#&^ | 0.2 ± 0.2^#&^ |
|  |  | No-vision | 15.5 ± 17.6^#&^ | 6.5 ± 6.9^#&^ |

Data are mean ± SD. Number sign (#) means a significant difference between force levels. Ampersand sign (&) denotes a significant difference between vision conditions. (*P* < 0.05).
