# Supplementary material for: Bilateral ankle dorsiflexion force control impairments in older adults
Source: PLoS One. 2025 Mar 20;20(3):e0319578. doi: 10.1371/journal.pone.0319578 (PMC11925285; doi:10.1371/journal.pone.0319578)
Supplement: S3 Table — MVC = maximum voluntary contraction, rRMSE = relative root mean square error, rBE- relative bias error, %CV = coefficient of variation, rcMSE = refined composite multiscale sample entropy. Asterisk (*) indicates P < 0.05. (DOCX) [file pone.0319578.s003.docx]

**S3 Table. Pearson's correlation between age and bilateral force control capabilities for the older group.**

|  | **Vision condition** | | | | **No-vision condition** | | | |
| --- | --- | --- | --- | --- | --- | --- | --- | --- |
|  | **10% MVC** | | **40% MVC** | | **10% MVC** | | **40% MVC** | |
| **Variable** | ***r*** | ***P*** | ***r*** | ***P*** | ***r*** | ***P*** | ***r*** | ***P*** |
| rRMSE | 0.055 | 0.794 | −0.241 | 0.245 | 0.308 | 0.135 | 0.015 | 0.942 |
| rBE | 0.178 | 0.393 | 0.155 | 0.458 | 0.241 | 0.246 | 0.116 | 0.579 |
| %CV | −0.020 | 0.923 | −0.193 | 0.355 | 0.227 | 0.275 | 0.251 | 0.226 |
| rcMSE | −0.432 | 0.031* | −0.385 | 0.057 | −0.335 | 0.102 | −0.409 | 0.043* |
| In-phase frequency | −0.161 | 0.442 | −0.257 | 0.214 | −0.172 | 0.411 | −0.265 | 0.200 |
| Anti-phase frequency | −0.202 | 0.333 | −0.070 | 0.739 | −0.174 | 0.415 | −0.012 | 0.954 |
| V_Index_ (Z-transform) | −0.118 | 0.575 | −0.320 | 0.119 | −0.031 | 0.885 | 0.013 | 0.951 |
| V_UCM_ | −0.113 | 0.592 | −0.195 | 0.349 | 0.262 | 0.206 | 0.121 | 0.563 |
| V_ORT_ | −0.034 | 0.870 | −0.055 | 0.793 | 0.331 | 0.106 | 0.137 | 0.513 |

*Abbreviations*. MVC = maximum voluntary contraction, rRMSE = relative root mean square error, rBE- relative bias error, %CV = coefficient of variation, rcMSE = refined composite multiscale sample entropy. Asterisk (*) indicates *P* < 0.05.
