# Supplementary material for: Bilateral ankle dorsiflexion force control impairments in older adults
Source: PLoS One. 2025 Mar 20;20(3):e0319578. doi: 10.1371/journal.pone.0319578 (PMC11925285; doi:10.1371/journal.pone.0319578)
Supplement: S1 Dataset — MVC = maximum voluntary contraction, rRMSE = relative root mean square error, rBE- relative bias error, %CV = coefficient of variation, rcMSE = refined composite multiscale sample entropy. (PDF) [file pone.0319578.s004.pdf]

S1 Dataset.

Vision 10% MVC									
Subject	rRMSE (% Target)	rBE (% Target)	%CV	rcMSE	In−phase	Anti−phase	V_{Index} (Z−transform)	V_{UCM}	V_{ORT}
Young 01	1.2	0.2	1.0	8.2	183.3	125.6	2.4	37.8	0.3
Young 02	1.0	0.6	0.7	7.9	138.2	108.3	1.0	0.8	0.1
Young 03	1.1	0.2	0.9	9.0	168.7	116.0	3.0	83.4	0.2
Young 04	2.6	1.4	1.8	6.8	211.7	141.9	2.0	28.9	0.6
Young 05	1.5	0.8	1.2	7.7	191.1	127.3	2.7	42.1	0.2
Young 06	1.1	−0.6	0.9	7.9	146.0	122.7	2.4	7.8	0.1
Young 07	1.6	−0.5	1.3	7.1	174.6	135.6	2.8	109.1	0.4
Young 08	1.7	−0.0	1.3	6.3	172.3	140.3	2.9	231.9	0.8
Young 09	1.6	−0.1	1.3	8.3	246.0	140.9	1.6	12.5	0.5
Young 10	1.7	0.2	1.6	7.4	185.4	146.0	2.4	17.1	0.1
Young 11	2.0	1.2	1.4	6.0	121.1	120.4	2.9	183.0	0.5
Young 12	2.0	0.0	1.8	8.1	248.4	155.1	2.4	40.1	0.3
Young 13	2.7	0.5	2.4	8.6	299.0	183.1	2.7	169.0	0.8
Young 14	2.1	−0.5	1.2	5.5	180.1	127.3	1.0	12.2	1.7
Young 15	2.8	2.1	1.7	8.1	286.1	176.9	2.4	25.2	0.2
Young 16	1.9	−0.8	1.6	9.3	406.0	188.4	1.9	6.5	0.1
Young 17	0.8	−0.2	0.8	10.6	341.0	129.9	3.9	107.3	0.0
Young 18	1.1	−0.1	1.0	7.3	248.2	139.7	3.6	94.1	0.1
Young 19	2.5	0.3	2.2	4.5	266.8	133.9	1.0	7.2	1.0
Young 20	1.7	0.5	1.3	8.2	336.4	157.9	2.5	85.4	0.6
Young 21	1.2	0.4	1.0	6.5	233.5	135.8	2.4	20.5	0.2
Young 22	1.0	0.3	0.9	8.5	234.4	134.9	3.8	95.1	0.1
Young 23	1.7	1.0	1.3	6.6	241.5	162.2	2.8	23.3	0.1
Young 24	1.5	0.3	1.4	7.9	327.0	152.4	2.8	26.6	0.1
Young 25	1.3	−0.3	1.2	10.4	349.4	178.5	1.4	41.3	2.5
Old 01	2.4	1.8	1.4	7.7	258.4	173.3	2.1	24.4	0.3
Old 02	2.2	0.2	2.1	8.0	329.1	178.8	3.3	145.2	0.2
Old 03	3.1	0.1	1.9	5.5	138.7	125.9	2.6	582.5	3.4
Old 04	1.1	0.6	0.9	9.1	137.3	100.0	1.5	4.2	0.2
Old 05	2.5	2.1	1.1	7.4	168.7	118.5	0.9	6.2	1.1
Old 06	2.4	1.9	1.4	5.3	203.0	115.9	2.7	81.8	0.3
Old 07	4.0	3.5	1.8	6.4	233.9	141.3	0.8	5.3	1.0
Old 08	1.6	0.8	1.2	9.0	357.4	148.0	2.1	17.3	0.3
Old 09	3.5	−0.1	2.2	4.8	150.8	108.4	1.8	168.6	4.7
Old 10	4.3	3.4	2.1	7.1	158.9	115.2	2.0	78.6	1.4
Old 11	2.3	0.4	1.8	4.4	179.6	115.6	1.6	25.1	1.0
Old 12	2.2	−1.1	1.6	5.5	168.3	123.6	0.8	3.4	0.7
Old 13	1.4	−0.3	1.2	6.6	165.3	126.0	2.2	19.9	0.2
Old 14	4.7	4.7	0.8	8.8	182.0	129.1	1.9	48.7	1.1
Old 15	1.7	0.6	1.3	6.6	237.9	131.1	2.4	71.2	0.6
Old 16	6.2	−1.6	5.6	6.6	375.0	130.0	1.5	69.3	3.6
Old 17	2.0	1.4	1.4	9.8	253.9	165.2	3.2	129.9	0.2

Old 18	2.1	−0.6	1.7	7.7	169.3	125.8	3.0	371.2	0.9
Subject	rRMSE (% Target)	rBE (% Target)	%CV	rcMSE	In−phase	Anti−phase	V_{Index} (Z−transform)	V_{UCM}	V_{ORT}
Old 19	3.0	0.3	2.9	7.3	284.4	135.0	1.6	13.1	0.6
Old 20	2.1	1.5	1.2	6.7	177.9	138.0	1.6	13.1	0.6
Old 21	4.6	3.8	2.4	8.2	417.4	194.3	1.7	21.4	0.7
Old 22	2.9	2.5	1.4	4.3	181.3	125.1	2.7	92.8	0.4
Old 23	2.9	0.7	2.7	5.1	231.4	145.0	2.3	68.9	0.7
Old 24	3.7	2.9	2.1	7.0	301.2	178.2	3.0	191.4	0.5
Old 25	4.0	2.0	2.5	5.3	226.2	139.8	2.4	137.7	1.2
**Vision 40% MVC**									
Young 01	1.1	−0.7	0.8	7.7	357.6	157.3	3.4	20.1	0.0
Young 02	1.1	−0.7	0.8	9.0	354.4	233.6	2.3	9.4	0.1
Young 03	1.1	−0.9	0.6	7.9	331.4	154.0	2.0	5.0	0.1
Young 04	1.5	−1.0	1.1	7.2	380.8	206.9	2.2	10.5	0.1
Young 05	2.5	−1.9	1.6	8.0	367.8	185.4	2.5	10.1	0.1
Young 06	2.9	−2.7	1.2	7.4	360.4	222.3	1.7	9.8	0.3
Young 07	1.9	−1.6	1.0	5.5	311.3	187.2	2.0	5.2	0.1
Young 08	2.4	−1.7	1.7	7.3	348.9	193.6	2.0	10.2	0.2
Young 09	3.5	−3.2	1.5	7.3	394.6	245.8	2.3	60.2	0.6
Young 10	2.5	−2.1	1.5	7.9	428.9	207.6	2.0	6.2	0.1
Young 11	2.6	−1.3	2.0	5.0	389.9	162.3	1.5	14.2	0.6
Young 12	2.0	−1.1	1.7	7.5	435.2	229.1	2.7	18.3	0.1
Young 13	3.2	−1.3	2.7	7.9	455.5	217.0	2.1	48.1	0.7
Young 14	4.0	−3.8	1.2	4.4	357.6	178.5	1.8	3.6	0.1
Young 15	2.0	−1.0	1.8	7.6	392.6	253.5	2.1	8.5	0.1
Young 16	2.0	−1.5	1.4	9.2	483.2	211.9	1.0	0.6	0.1
Young 17	1.7	−1.4	0.9	10.0	493.4	220.6	2.0	5.4	0.1
Young 18	2.6	−2.3	1.2	8.4	436.3	200.8	1.4	3.5	0.2
Young 19	2.3	−1.6	1.6	6.4	411.9	218.1	2.1	8.4	0.1
Young 20	2.0	−1.5	1.4	7.9	429.9	252.7	1.8	6.7	0.2
Young 21	1.2	−0.6	1.1	7.7	392.1	256.8	2.2	9.1	0.1
Young 22	1.2	−0.7	0.9	7.8	398.5	255.3	3.4	12.9	0.0
Young 23	1.9	−1.3	1.4	8.0	419.3	252.8	1.8	5.0	0.1
Young 24	1.4	−0.9	1.1	7.5	408.9	231.8	1.8	2.9	0.1
Young 25	1.5	−1.1	1.0	10.7	513.5	205.9	1.6	14.3	0.6
Old 01	1.8	−0.8	1.2	7.5	404.5	246.2	1.1	5.2	0.6
Old 02	1.8	−0.8	1.6	8.7	441.9	227.3	2.8	16.0	0.1
Old 03	4.1	−3.9	1.3	7.5	411.0	144.7	1.9	41.1	1.0
Old 04	1.8	−1.1	1.4	8.7	444.1	159.0	2.3	22.8	0.2
Old 05	1.4	−0.8	1.2	6.9	430.0	169.4	1.8	3.1	0.1
Old 06	1.2	0.2	1.1	5.3	429.5	152.8	2.2	5.6	0.1
Old 07	4.2	−3.9	1.6	7.4	439.3	196.8	2.1	12.1	0.2
Old 08	1.7	−1.2	1.2	7.7	394.5	221.8	2.1	6.4	0.1
Old 09	3.1	−3.0	1.0	5.9	253.9	127.0	1.3	5.4	0.4
Old 10	1.8	0.6	1.5	5.9	325.4	153.2	2.3	37.1	0.4

Old 11	2.6	−1.8	1.8	5.4	390.6	193.7	2.1	14.5	0.2
Old 12	3.6	−2.9	2.0	4.1	407.7	115.9	1.0	3.2	0.5
Subject	rRMSE (% Target)	rBE (% Target)	%CV	rcMSE	In−phase	Anti−phase	V_{Index} (Z−transform)	V_{UCM}	V_{ORT}
Old 13	1.9	−1.6	1.0	6.3	391.1	144.3	1.7	7.7	0.3
Old 14	4.0	3.7	1.1	7.8	458.9	208.1	2.2	38.2	0.5
Old 15	2.3	−1.5	1.7	5.8	517.6	131.6	3.1	90.6	0.2
Old 16	4.4	−2.4	3.6	6.9	498.3	158.9	1.6	23.4	0.9
Old 17	1.6	−0.5	1.4	8.2	373.9	231.2	2.8	70.9	0.3
Old 18	4.6	−3.8	2.5	4.7	383.8	172.0	1.7	53.3	1.8
Old 19	3.4	−1.9	2.8	8.3	620.0	103.9	3.0	22.7	0.1
Old 20	2.9	−2.0	2.2	6.1	490.2	144.8	2.0	22.9	0.4
Old 21	1.8	0.4	1.6	6.8	419.9	263.8	2.2	23.0	0.3
Old 22	1.8	0.5	1.4	3.3	357.6	156.0	1.2	6.5	0.6
Old 23	2.0	−0.4	1.9	6.1	437.0	192.8	2.7	95.6	0.4
Old 24	1.6	−0.9	1.3	8.6	491.9	218.1	3.2	28.7	0.0
Old 25	2.5	−1.7	1.9	5.2	353.7	191.3	2.2	23.5	0.3
**No−vision 10% MVC**									
Young 01	4.7	3.9	2.0	4.4	165.3	124.7	0.8	27.1	5.7
Young 02	3.1	2.0	1.6	3.5	144.3	113.0	0.5	11.2	3.9
Young 03	5.0	3.9	1.8	4.6	167.2	121.7	0.2	14.8	10.6
Young 04	5.6	2.7	3.2	3.6	205.6	150.6	0.6	32.0	9.2
Young 05	5.1	4.1	2.4	3.7	164.4	151.3	0.3	9.5	5.0
Young 06	4.7	4.2	1.8	5.9	148.5	128.6	0.1	8.3	6.6
Young 07	5.7	2.5	2.4	4.4	186.6	147.5	1.0	119.4	17.1
Young 08	7.0	2.1	3.1	3.6	197.0	162.2	0.5	69.6	27.0
Young 09	7.2	2.8	3.4	3.3	208.5	147.6	0.0	21.6	20.6
Young 10	4.0	−2.5	2.3	5.5	199.9	153.9	0.5	8.7	3.1
Young 11	6.0	4.8	2.1	4.2	132.6	120.9	1.0	56.5	7.0
Young 12	10.8	−0.4	7.4	2.0	240.2	157.9	1.0	307.4	39.0
Young 13	15.6	−10.4	7.8	4.0	295.2	220.7	−0.1	64.6	87.2
Young 14	3.9	1.4	1.9	3.6	160.9	130.2	−0.8	1.5	7.1
Young 15	11.9	10.5	4.1	4.3	292.9	190.6	−0.3	10.5	18.5
Young 16	4.2	1.8	2.7	6.5	389.3	205.8	0.2	10.4	7.1
Young 17	3.7	2.6	1.9	6.6	354.1	169.9	1.0	22.5	2.9
Young 18	3.5	0.8	2.2	5.4	306.4	176.2	0.4	12.0	5.7
Young 19	7.3	4.8	4.2	2.1	236.8	147.0	1.0	65.1	9.2
Young 20	7.4	6.7	2.4	4.3	317.4	174.8	1.0	64.6	8.3
Young 21	4.9	−4.1	2.3	3.8	266.4	156.8	0.4	17.4	7.5
Young 22	5.7	0.6	3.4	3.7	252.2	151.5	−0.2	17.2	23.4
Young 23	6.9	0.4	4.0	3.8	289.6	188.0	0.6	78.6	25.1
Young 24	4.4	0.4	3.2	5.3	357.3	188.1	0.9	28.5	5.2
Young 25	4.1	−1.0	2.5	6.8	357.3	193.9	0.1	30.5	24.8
Old 01	16.7	15.5	5.1	1.3	223.7	172.5	1.0	65.7	9.4
Old 02	8.0	0.9	4.4	4.8	334.5	185.3	0.0	28.0	29.3
Old 03	10.1	8.4	4.3	2.9	180.8	138.8	0.8	95.4	21.0

Old 04	6.3	5.2	2.4	3.8	106.6	93.4	−0.8	2.9	13.6
Old 05	6.8	6.4	2.0	4.4	165.8	125.0	0.6	8.3	2.4
Old 06	7.3	3.8	3.6	2.5	197.2	144.6	1.2	174.9	16.5
Subject	rRMSE (% Target)	rBE (% Target)	%CV	rcMSE	In−phase	Anti−phase	V_{Index} (Z−transform)	V_{UCM}	V_{ORT}
Old 07	17.8	16.7	5.2	2.0	216.4	147.5	0.1	16.4	14.5
Old 08	17.8	16.2	6.3	1.3	316.1	181.8	−0.1	9.4	12.1
Old 09	6.9	−5.7	3.3	3.3	141.3	116.5	0.6	19.2	5.3
Old 10	10.1	9.1	3.6	4.6	163.1	112.1	−0.3	18.3	34.4
Old 11	7.1	5.2	3.7	3.1	189.3	130.5	−0.7	5.1	19.6
Old 12	6.3	2.0	2.9	3.8	177.0	128.6	0.2	31.5	19.9
Old 13	7.9	7.5	1.9	4.3	178.1	119.2	1.0	53.7	6.9
Old 14	15.5	14.8	3.9	2.2	219.8	153.5	0.5	25.4	9.3
Old 15	8.9	6.4	3.4	3.8	245.6	137.2	0.7	173.6	43.8
Old 16	14.7	13.3	4.6	6.1	283.1	149.9	0.8	100.9	18.6
Old 17	13.6	11.8	5.5	4.7	277.6	216.4	1.0	206.2	29.8
Old 18	16.0	13.5	6.7	2.2	179.2	127.1	0.0	43.4	46.9
Old 19	9.6	8.1	4.3	3.8	241.5	136.5	0.3	21.3	12.7
Old 20	7.7	6.9	2.6	3.2	164.2	131.7	0.1	13.8	10.3
Old 21	23.0	21.4	6.6	2.1	318.0	222.2	0.3	51.4	30.7
Old 22	24.1	22.0	7.5	0.7	207.9	149.3	0.1	66.0	54.3
Old 23	9.4	2.2	4.7	3.8	242.4	143.6	1.3	734.5	55.2
Old 24	5.1	0.8	2.8	5.1	296.0	201.0	1.1	135.6	14.1
Old 25	10.2	−0.7	3.1	4.1	179.8	145.4	−0.4	31.7	66.1
**No−vision 40% MVC**									
Young 01	3.5	−2.7	1.7	4.0	275.5	208.1	0.3	6.6	3.5
Young 02	5.7	−5.1	2.6	3.7	287.6	236.8	0.8	18.4	3.7
Young 03	4.6	−4.0	2.2	2.7	295.6	180.6	1.0	20.1	2.9
Young 04	4.8	−4.2	2.4	3.4	346.9	229.1	0.9	10.5	1.7
Young 05	12.8	−12.0	5.1	1.9	311.5	225.3	0.7	9.6	2.2
Young 06	7.3	−6.8	2.8	4.2	362.2	220.6	0.0	5.2	5.4
Young 07	7.3	−6.6	3.2	1.9	313.7	217.6	0.2	3.2	2.2
Young 08	6.0	−4.9	3.3	4.2	304.5	203.0	0.3	10.1	5.0
Young 09	12.3	−11.7	4.2	2.0	372.6	248.3	2.0	29.4	0.6
Young 10	14.1	−13.1	5.9	2.0	325.9	265.1	0.0	2.7	2.7
Young 11	9.7	−7.2	5.3	2.4	380.9	180.8	−0.9	3.1	18.0
Young 12	7.3	−3.7	5.0	2.7	399.6	251.4	−0.2	9.8	13.6
Young 13	11.1	−7.4	7.5	4.2	377.1	269.7	0.0	36.2	33.4
Young 14	7.3	−6.9	2.7	2.1	351.1	176.6	−0.1	5.8	6.5
Young 15	8.9	−5.9	4.9	3.2	358.0	267.0	0.4	8.3	4.1
Young 16	4.5	0.3	3.2	5.6	433.1	262.7	−0.7	1.0	4.5
Young 17	5.1	−4.7	2.0	5.4	424.0	263.7	0.0	1.8	1.9
Young 18	8.0	−7.0	4.0	3.3	427.8	221.8	−0.8	1.6	7.9
Young 19	12.7	−11.8	5.1	1.3	398.3	236.5	−0.4	3.7	9.0
Young 20	6.3	−5.6	2.7	4.1	404.8	263.7	−0.2	3.9	5.6
Young 21	8.5	−7.7	4.0	1.5	375.0	236.9	1.0	8.6	1.1

Young 22	8.7	−8.1	3.5	1.4	371.6	257.8	−0.2	3.6	5.4
Young 23	10.2	−9.1	4.8	3.1	372.2	284.7	−0.3	2.7	4.8
Young 24	3.6	−2.4	1.8	6.1	403.7	251.8	−0.5	2.3	5.6
Young 25	9.5	−8.6	4.3	2.8	438.3	240.4	−0.2	7.9	11.8
Subject	rRMSE (% Target)	rBE (% Target)	%CV	rcMSE	In−phase	Anti−phase	V_{Index} (Z−transform)	V_{UCM}	V_{ORT}
Old 01	5.5	−5.2	2.0	4.3	386.4	251.0	0.8	23.3	5.2
Old 02	11.2	−7.4	4.6	2.9	405.2	258.0	−0.3	20.4	35.3
Old 03	10.5	−9.7	4.2	1.8	305.4	175.5	0.5	24.4	9.3
Old 04	4.9	−3.8	2.5	5.1	458.6	154.6	−0.1	5.3	6.5
Old 05	3.8	−0.3	2.2	4.2	387.8	180.7	0.2	11.5	7.1
Old 06	4.5	−1.8	2.3	2.7	391.6	190.5	0.4	21.6	9.0
Old 07	12.6	−11.7	5.2	1.5	384.6	241.8	0.2	5.9	3.7
Old 08	5.4	−4.1	2.4	3.2	378.9	223.3	−0.1	6.5	8.1
Old 09	9.6	−9.2	2.9	1.9	244.4	142.9	0.7	8.5	2.0
Old 10	3.7	0.5	1.6	4.5	278.0	146.9	0.6	24.8	6.9
Old 11	12.0	−11.3	4.5	1.5	357.0	201.9	0.0	5.5	5.5
Old 12	4.2	0.1	2.8	3.2	429.0	135.1	−0.2	5.6	9.1
Old 13	8.1	−7.7	2.8	2.9	365.0	178.8	0.2	7.2	5.3
Old 14	5.0	4.5	1.8	4.3	392.3	222.9	0.4	4.8	2.2
Old 15	6.2	−5.0	2.8	4.2	470.9	163.5	−0.2	11.3	16.6
Old 16	15.6	−14.5	6.6	3.1	453.4	192.2	0.1	24.8	20.0
Old 17	9.4	−8.6	4.2	3.9	362.5	265.1	−0.5	2.4	7.0
Old 18	4.7	2.1	2.3	4.4	346.1	147.7	−0.6	2.8	10.2
Old 19	19.5	−18.5	7.4	2.6	486.7	155.5	−0.7	3.4	13.9
Old 20	6.0	−3.9	2.6	4.3	424.8	179.7	0.2	26.1	16.7
Old 21	4.3	−3.1	2.3	4.1	410.3	274.4	0.6	12.9	3.5
Old 22	8.9	−4.9	6.5	1.3	327.5	181.2	−0.7	8.1	30.0
Old 23	8.6	6.8	4.2	3.5	365.2	246.0	0.3	27.5	15.3
Old 24	5.7	−5.0	2.7	5.2	429.9	242.8	0.7	18.8	4.6
Old 25	13.6	−12.9	4.7	1.8	356.1	196.0	0.2	4.7	2.9

Abbreviations. MVC = maximum voluntary contraction, rRMSE = relative root mean square error, rBE− relative bias error, %CV = coefficient of variation, rcMSE = refined composite multiscale sample entropy.
